# Gastrointestinal symptoms as an extended clinical feature of Pierson syndrome: a case report and review of the literature

**DOI:** 10.1186/s12881-020-01019-9

**Published:** 2020-04-15

**Authors:** Kei Nishiyama, Mari Kurokawa, Michiko Torio, Yasunari Sakai, Mitsuru Arima, Shoko Tsukamoto, Satoshi Obata, Shogo Minamikawa, Kandai Nozu, Noriyuki Kaku, Yoshihiko Maehara, Koh-Hei Sonoda, Tomoaki Taguchi, Shouichi Ohga

**Affiliations:** 1 Department of Pediatrics, Graduate School of Medical Sciences, Kyushu University, 3-1-1 Maidashi, Higashi-ku, Fukuoka, 812-8582 Japan; 2 Department of Ophthalmology, Graduate School of Medical Sciences, Kyushu University, Fukuoka, Japan; 3 Department of Pediatric Surgery, Graduate School of Medical Sciences, Kyushu University, Fukuoka, Japan; 4 Department of Pediatrics, Kobe University Graduate School of Medicine, Kobe, Japan; 5 Emergency and Critical Care Center, Kyushu University Hospital, Fukuoka, Japan

**Keywords:** Pierson syndrome, The laminin β2 gene (*LAMB2*), Microcoria, Neurological signs, intestinal malrotation, Autonomic dysfunction

## Abstract

**Background:**

Pierson syndrome (PS) is a rare autosomal recessive disorder, characterized by congenital nephrotic syndrome and microcoria. Advances in renal replacement therapies have extended the lifespan of patients, whereas the full clinical spectrum of PS in infancy and beyond remains elusive.

**Case presentation:**

We present the case of a 12-month-old boy with PS, manifesting as the bilateral microcoria and congenital nephrotic syndrome. He was born without asphyxia, and was neurologically intact from birth through the neonatal period. Generalized muscle weakness and hypotonia were recognized from 3 months of age. The infant showed recurrent vomiting at age 5 months of age, and was diagnosed with gastroesophageal reflux and intestinal malrotation. Despite the successful surgical treatment, vomiting persisted and led to severely impaired growth. Tulobuterol treatment was effective in reducing the frequency of vomiting. Targeted sequencing confirmed that he had a compound heterozygous mutation in *LAMB2* (NM_002292.3: p.Arg550X and p.Glu1507X). A search of the relevant literature identified 19 patients with severe neuro-muscular phenotypes. Among these, only 8 survived the first 12 months of life, and one had feeding difficulty with similar gastrointestinal problems.

**Conclusions:**

This report demonstrated that severe neurological deficits and gastrointestinal dysfunction may emerge in PS patients after the first few months of life.

## Background

Pierson syndrome (PS) is a rare autosomal recessive disorder, characterized by congenital nephrotic syndrome and microcoria [1]. These phenotypes are caused by the mutations of *LAMB2*, which encodes laminin β2 [2]. Among various isoforms, Laminin-521 is known to be the most common isoform containing the β2 subunit [3]. In accordance with the expression of *LAMB2* at the neuromuscular junctions, patients with PS show severe muscular hypotonia and developmental delay [2].

The majority of patients with PS die of renal failure within the first year of life. Despite this vital symptom, recent evidence has shown that the implementation of renal replazcement therapy in infancy is effective for extending life span [4, 5]. On the other hand, the full clinical spectrum of the long-term survivors and the time course of neuromuscular complications remain elusive. In this report, we present the case of a patient with PS carrying a compound heterozygous mutation in *LAMB2*. The patient showed progressive gastrointestinal as well as dysautomomic features as novel complications of PS.

## Case presentation

The patient, a 12-month-old boy was the third child of non-consanguineous, healthy parents. He had healthy sisters of 5 and 4 years of age. The patient was born via vaginal delivery without asphyxia at 35 weeks of gestational age. The patient’s birth weight, length and head circumference were 2342 g (0.0 SD), 45.2 cm (0.0 SD) and 33.4 cm (1.2 SD), respectively. The placental weight was 850 g. Oliguria continued from birth, and the serum creatinine level was elevated to 2.63 mg/dl on the 5th day of age. Congenital nephrotic syndrome was suspected based on the detection of hypoalbuminemia (2.1 g/dl) and proteinuria (3+ on dipstick).

He was transferred to our hospital at 6 days of age. On admission, his consciousness was alert and his vital signs were stable: respiratory rate 37/min, heart rate 149/min, and blood pressure 84/54 mmHg. Bilateral microcoria and nystagmus were evident. The body posture and voluntary movements were normal. The muscular tone appeared normal. Deep tendon reflex was brisk. Laboratory tests confirmed that the urine protein/creatinine ratio was increased to 95.1 mg/mg Cre. Renal ultrasonography showed hyperechoic signals in both kidneys with unclear cortico-medullary structures. Continuous hemodiafiltration was introduced at 7 days of age, which was then replaced by peritoneal dialysis from 12 days of age. He became anuric during the course and continued peritoneal dialysis afterwards. With these renal replacement therapies, he survived the first 12 months of life.

**Fig. 1:**
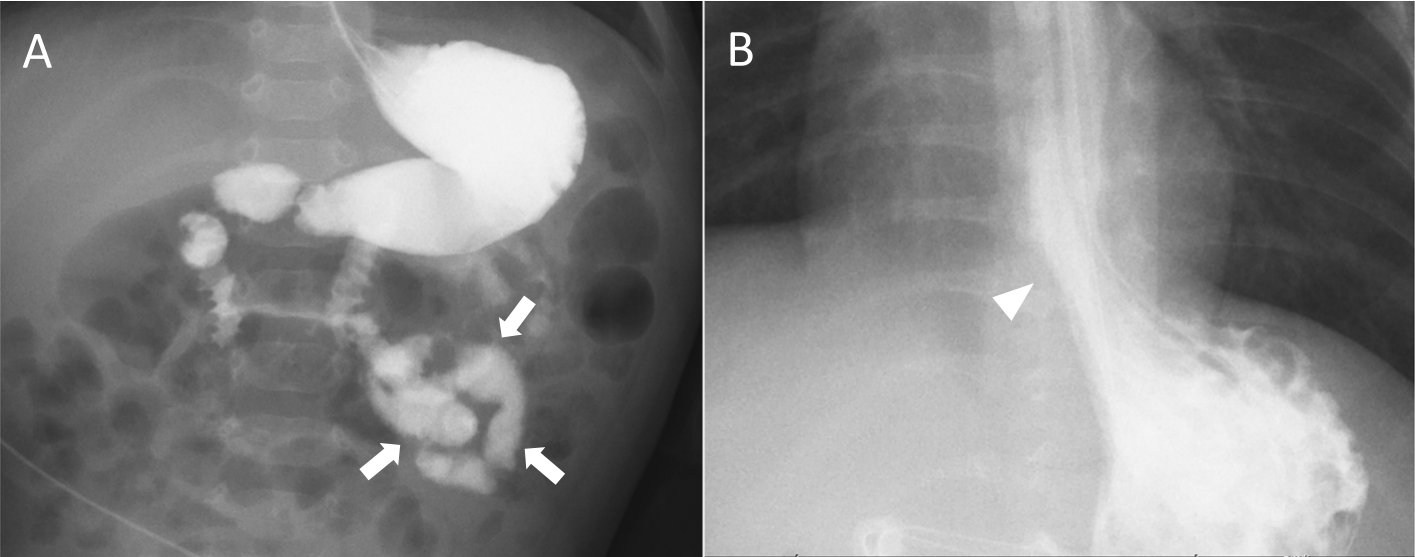
Gastrointestinal phenotypes associated with Pierson syndrome. **a** An upper gastrointestinal contrast study depicting the intestinal malrotation. Arrows indicate the “corkscrew sign” of the proximal jejunum. **b** A radiographic contrast image depicting the gatroesophageal reflux (arrow head)

Intestinal malrotation was diagnosed after the patient began experiencing recurrent vomiting started at 6 months of age (Fig. 1a). The volvulus was successfully relieved by surgery. However, the vomiting signs persisted beyond the surgical repair due to the presence of another digestive dysfunction, gastroesophageal reflux (Fig. 1b). Although duodenal tube feeding was introduced, only limited amounts of nutrition could be provided, which proved insufficient to achieve weight gain during the 12-month follow-up period. Thus, tulobuterol (0.2 mg/day) was given orally from 12 months. The patient’s vomiting disappeared immediately after tulobuterol treatment, and his weight continued to increase until the time of writing this report. In the last month, it reached 5300 g (+ 300 g). Patients on peritoneal dialysis are also known to develop thyroid dysfunctions [6]. We verified that serum thyroid stimulating hormone (2.70 μIU/mL) and free thyroxine (1.48 ng/dL) levels remained unaltered. We thus did not interpret hyper- or hypothyroidism as a primary cause of his gastrointestinal problems.

Serial neurological assessments demonstrated that he had started gazing at and pursuing the objects from 2 months of age. However, generalized muscle weakness and hypotonia became prominent from 3 months of age. Presently at 12 months of age, his developmental quotient score is considered to be less than 20 (equivalent to the score of an infant of 2–3 months of age). He has been unable to control his neck, roll over, or sit unaided. Electrocardiography showed normal sinus rhythm and a normal axis with a corrected QT interval of 356 msec. The coefficient of variation in electrocardiographic R-R intervals (13.5%) remained within the normal range. Echocardiography demonstrated the balance of the 4 chambers and a normal left-heart function with an ejection fraction of 82.9%.

**Fig. 2:**
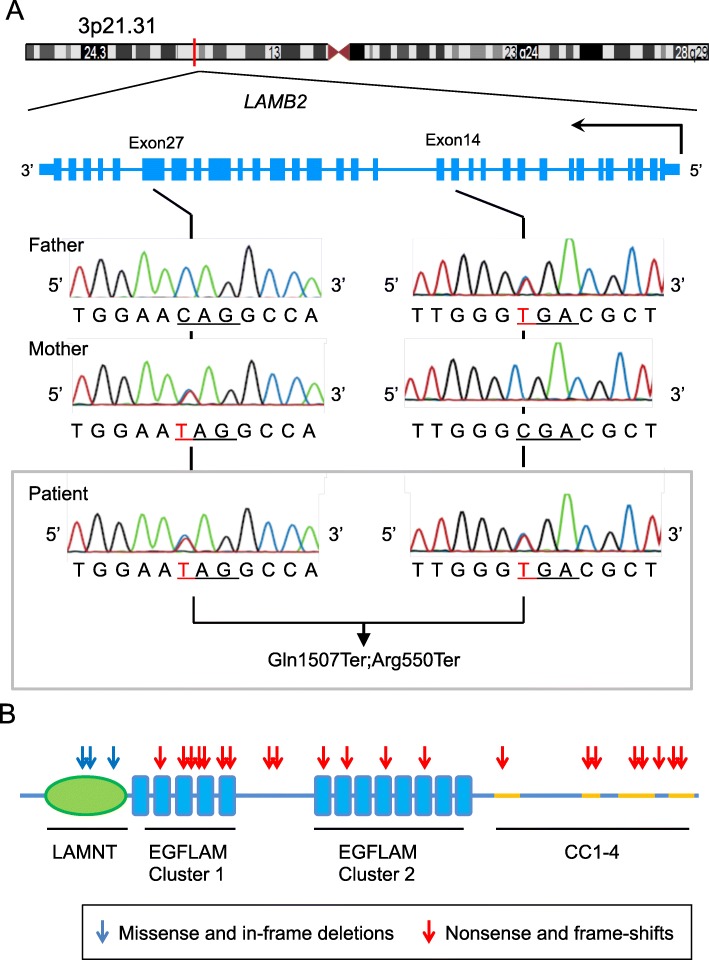
*LAMB2* mutations in this and previously reported cases of Pierson syndrome. **a** Sequencing chromatograms of the father, mother and the patient are shown. The 5′ to 3′ ends of the transcripts are indicated in combination with each diagram. Vertical arrows indicate the position of mutations; exon14 (c.1648C > T:p.Arg550X) and exon 27 (c.4519C > T:p.Glu1507X), and mutated codons are underlined. **b** Pathogenic mutations associated with the neuromuscular symptoms of PS are mapped to the functional domains of Laminin β2 protein. Red arrows: nonsense and frame-shift mutations; Black arrows: missense mutations and in-frame deletions. LAMNT, laminin N-terminal globular domain; EGFLAM, laminin EGF-like modules; CC, laminin coiled coil domain

The clinical features of congenital nephrotic syndrome with microcoria prompted us to conduct the genetic testing for the diagnosis of PS. Whole-exome sequencing followed by the Sanger method showed that the patient harbored a compound heterozygous mutations in exon 14 (NM_002292.3:c.1648C > T:p.Arg550X) and exon 27 (NM_002292.3:c.4519C > T:p.Glu1507X) in the *LAMB2* gene (Fig. 2a). An inheritance analysis demonstrated that his father and mother carried the heterozygous mutations in exon 14 (NM_002292.3:c.4519C > T:p.Glu1507X) and exon 27 (NM_002292.3:c.4519C > T:p.Glu1507X), respectively. Thus, the patient was genetically diagnosed with PS.

We found 19 patients with PS, who have been reported to manifest severe neuro-muscular problems (Table 1). All of these patients showed neurological signs of hypotonia or developmental delay, whereas their onset or time course was not described in detail. Eight patients survived 12 months after birth, and 7 of them received renal replacement therapy (dialysis or kidney transplantation) in early infancy. Among them, only one patient was reported to show gastrointestinal problems. No detailed information was available on the gastrointestinal features of the other long-term survivors.

**Table 1:** Summary of the patients with neuromuscular phenotypes of Pierson syndrome

Case	Author	Year	Mutation	Sex	Age at the report (Status)	Renal disease (Age at onset)	Neurological finding	Ophthalmology	Gastrointestinal symptom	Renal replacementtherapy (Age)
1	Maselli RA	2009	p.C493SfsTer;p.Q1602RfsTer52	F	20 years	ProteinuriaESRD(Birth)	Hypotonia, Motor delay	Microcoria	NA	Kidney transplant (15 months)
2	Wühl E	2007	p.Q1753fsTer7(homozygous)	F	19 months (Died)	ProteinuriaESRD(Birth)	Hypotonia, Motor delay,Cognitive deficits, Microcephaly	Microcoria, Cataract, PHPV	Feeding difficulty;gastrostomy	Peritoneal dialysis (6 weeks)Kidney transplant(18 months)
3	Zenker M	2004	p.E1754GfsTer7(homozygous)	NA	19 months (Died)	ProteinuriaESRD(< 1 m)	Hypotonia, Motor delay	Abnormal lens	NA	Peritoneal dialysis
4	Wühl E	2007	p.C493fsTer3;p.E1301fsTer57	F	17 months	ProteinuriaESRD(Birth)	Hypotonia, Motor delay, Cognitive deficits, Microcephaly	Microcoria, Cataract, PHPV	NA	Peritoneal dialysis (7 weeks)
5	Matejas V	2011	p.R468fsTer37[Intron 10 c.1405 + 1G > A] splicedonor site, paternal isodisomy ofCh3	M	17 months (Died of aspergillosis)	ProteinuriaESRD(Birth)	Hypotonia, Motor delay, Cognitive deficits	Microcoria, Retinal detachment	NA	Peritoneal dialysis (3 weeks)
6	Wühl E	2007	p.M415fsTer81;p.Q418Ter	F	15 months (Died of sepsis)	ProteinuriaESRD(Birth)	Hypotonia, Motor delay, Cognitive deficits, Microcephaly	Microcoria, Cataract, Retinal detachment,PHPV	NA	Peritoneal dialysis (3 weeks)
7	Bredrup C	2008	p.R246W;p.R575Ter	M	15 months	ProteinuriaESRD(1w)	Hypotonia, Motor delay, Cognitive deficits, Microcephaly	Microcoria, Abnormal lens	NA	NA
8	This report	2018	p.R550Ter;p.Q1507Ter	M	12 months	ProteinuriaESRD(Birth)	Hypotonia,Motor delay	Microcoria	Intestinalmalrotation, Severe gastroesophageal reflux	Hemodialysis (7 days) Peritoneal dialysis (12 days)
9	Matejas V	2010	p.L139P(homozygous)	NA	12 months (Died)	Proteinuria(3 m)	Hypotonia, Motor delay,Cognitive deficits	Microcoria, Abnormal lens	NA	NA
10	Zenker M	2004	p.R246W(homozygous)	M	8 months(Died of sepsis)	Proteinuria(5 m)	Hypotonia, abnormal movement ofright arm	Microcoria	NA	NA
11	Zenker M	2004	p.R246W(homozygous)	F	8 months(Died of metaboliccause and infection)	Proteinuria(5d)	Hypotonia, Left hemiparesis	Microcoria, Cataract	NA	Peritoneal dialysis (4 months)
12	Matejas V	2010	p.L139P(homozygous)	NA	5 months (Died)	Proteinuria(<1w)	Hypotonia, Motor delay	Abnormal lens	NA	NA
13	Bredrup C	2008	p.Q868Ter;p.C1058Ter	M	4.5 months	ProteinuriaESRD(2w)	Hypotonia	Microcoria	NA	NA
14	Bredrup C	2008	p.V808WfsTer342(homozygous)	M	4 months (Died)	ProteinuriaESRD(1w)	Hypotonia, Motor delay, Microcephaly	Microcoria	NA	NA
15	Bredrup C	2008	p.I149del;p.G1693VfsTer20	M	2 months (Died)	ProteinuriaESRD(1w)	Hypotonia	Microcoria	NA	NA
16	Matejas V	2010	p.E1636AfsTer22(homozygous)	NA	1.2 months (Died)	ProteinuriaESRD(1w)	Hypotonia,Motor delay,Microcephaly	Microcoria, Abnormal lens	NA	NA
17	Bredrup C	2008	p.L627AfsTer4;p.R1502GfsTer17	M	1 month (Died)	ProteinuriaESRD(1w)	Hypotionia	Microcoria	NA	
18	Zenker M	2005	p.C374X;p.Y689X	NA	2 weeks (Died)	ProteinuriaESRD(1w)	Hypotinia	Microcoria, Abnormal lens,	NA	NA
19	Zemrani B	2016	p.R964X(homozygous)	M	7 days (Died)	ProteinuriaESRD(Birth)	Hypotonia	Microcoria	NA	Peritoneal dialysis (birth)

*NA* data not available

Mutation maps of these 19 patients are graphically summarized in Fig. 2b. Notably, all truncating mutations were located outside the laminin N-terminal (LAMNT) globular domain and the missense or in-frame deletions were mapped to the LAMNT domain. This result indicated both the pathogenic impacts of the disrupted LAMB2 protein and the functional significance of the LAMNT domain.

## Discussion and conclusions

We reported the case of a patient with PS carrying truncating mutations in *LAMB2*. This report extended the phenotypic spectrum of PS by clarifying that gastrointestinal dysfunction emerged after the first few months of life. Earlier studies have demonstrated the clinical course of patients with PS, and clarified their pathological bases in both human subjects and animal models. In agreement with the clinical course of the present case, a previous report supports the notion that neuromuscular deficits may emerge and progress in early infancy [5, 7–9]. In animal studies, the morphological analyses revealed that the number of presynaptic vesicles and the size of nerve termini at the neuro-muscular junctions (NMJ) did not differ between *Lamb2*-nulli mice and their littermates during the first few days of life [10]. Notably, however, prominent differences emerged after postnatal day 7 [11]. These findings suggest that laminin β2 is important for postnatal maturation of NMJ in both humans and mice during the first few weeks after birth. In this regard, the absence of neurological findings at birth does not imply that patients with PS will show mild symptoms in late infancy.

In addition to the detailed time course of the neurological findings, this report first disclosed that PS patients may develop intestinal malrotation and gastro-esophageal reflux. Notably, we found a report presenting the case of a patient with PS, who survived the neonatal period and underwent gastrostomy due to poor feeding [12]. Experimental data also showed that laminin β2 is expressed in upper gastrointestinal tract [13]. In some PS patients, neuromuscular symptoms showed initial improvement with the administration of oral ephedrine [5]. The mode of action of β-stimulants is not fully understood; however, they increase the release of quanta of acetylcholine and reduce the acetylcholine receptor open time in vitro [14, 15]. Tulobuterol possibly acts at the autonomic nerve termini, which might have contributed to the amelioration of the gastrointestinal symptoms of this patient. These findings suggest that the gastrointestinal dysfunction is not a rare complication in patients with PS.

Intestinal malrotation represents a heterogeneous group of disorders, and the genetic backgrounds for this complex condition remain to be explored [16]. Nonetheless, 4 main factors have been proposed to explain its underlying mechanisms: 1) abnormal left-right patterning, 2) an aberrant dorsal mesentery, 3) dysfunction of the intestine itself, or 4) dysfunction of other abdominal contents [17]. Given the expression of *LAMB2* in the intestine, the third mechanism is likely to support the pathogenic link between PS and the intestinal malrotation in the present case. The correlations of the timeframe of the appearance of neurological signs and the intestinal malrotations will be explored more extensively in future studies with additional cases with PS.

This is the first report to delineate the time course of neurological signs and gastrointestinal involvements in a patient with PS, as well as the extended spectrum of the clinical phenotype.

## Data Availability

All data are available in this manuscript and supplementary information. The datasets generated during the current study are available at the ClinVar (www.ncbi.nlm.nih.gov/clinvar/).

## Abbreviations

PS: Pierson syndrome
LAMNT: Laminin N-terminal
NMJ: neuro-muscular junctions
